# Dietary Acid Load: A Novel Nutritional Target in Overweight/Obese Children with Asthma?

**DOI:** 10.3390/nu11092255

**Published:** 2019-09-19

**Authors:** Pedro Cunha, Inês Paciência, João Cavaleiro Rufo, Francisca Castro Mendes, Mariana Farraia, Renata Barros, Diana Silva, Luís Delgado, Patrícia Padrão, André Moreira, Pedro Moreira

**Affiliations:** 1 Faculdade de Ciências da Nutrição e Alimentação da Universidade do Porto, 4200-465 Porto, Portugal; pedrocunha27@hotmail.com(P.C.); renatabarros@fcna.up.pt (R.B.); Patriciapadrao@fcna.up.pt (P.P.); andremoreira@med.up.pt (A.M.); 2 Imunologia Básica e Clínica, Departamento de Patologia, Faculdade de Medicina, Universidade do Porto, 4200-319 Porto, Portugal; inespaciencia@gmail.com (I.P.); francisca_castromendes@hotmail.com (F.C.M.); disolha@gmail.com (D.S.); ldelgado@med.up.pt (L.D.); 3 EPIUnit Instituto de Saúde Pública, Universidade do Porto, 4050-600 Porto, Portugal; jcrufo@gmail.com (J.C.R.); mariana.farraia@gmail.com (M.F.); 4 Institute of Science and Innovation in Mechanical Engineering and Industrial Management (INEGI), 4200-465 Porto, Portugal; 5 Serviço de Imunoalergologia, Centro Hospitalar Universitário de São João, 4200-319 Porto, Portugal

**Keywords:** asthma, obesity, dietary acid load, PRAL

## Abstract

Obesity has been repeatedly linked to asthma, and several potential mechanisms have been proposed in the etiologies of the obese-asthma phenotype. Considering that lungs play an important role in systemic pH and acid–base regulation, are a key organ in asthma development, and that nutritional inadequacy of several nutrients and high dietary acid load can affect airway inflammation and reactivity, we aimed to test the hypothesis that dietary acid load may be associated with asthma in children. Data on 699 children (52% females), aged 7–12 years, were analyzed. Anthropometric measurements were performed to assess body mass index. Dietary acid load was calculated using potential renal acid load (PRAL) equations from a 24 h dietary recall administrated to children. Adjusted PRAL for total energy intake was applied with the use of the residual method. Lung function and airway reversibility were assessed with spirometry. Asthma was defined by a positive bronchodilation or self-reported medical diagnosis with reported symptoms (wheezing, dyspnea, or dry cough) in the past 12 months. After adjustment for energy intake, sex, age, parent’s education level, and physical activity, positive and significant associations were found between asthma and PRAL [odds ratio (OR) = 1.953, 95% CI = 1.024, 3.730) in overweight/obese children. Our findings suggest that dietary acid load might be a possible mechanism in overweight/obese-asthma phenotype development.

## 1. Introduction

Asthma is a chronic inflammatory pathology of the airways that is defined by the association between inflammation and airway hyperresponsiveness that leads to recurrent episodes of wheezing, breathlessness, chest tightness, and coughing. These episodes also can be present with airflow obstruction within the lung, which is often reversible either spontaneously or with treatment [1]. Asthma has been repeatedly linked to obesity [2], and these two chronic conditions may share genetic and lifestyle drivers, such as physical activity and nutritional and dietary factors [3,4,5]. Obesity is one of the main public health problems [6]. In the last thirty years, the prevalence of obesity increased by 47% in children worldwide as a result of a complex relationship between genetic, socioeconomic and cultural factors, consumption patterns, urban development, and lifestyle habits [7]. In Portugal, overweight and obesity prevalence in children is around 8–9% and 17%, respectively [8]. However, the etiologies of obese-asthma and the mechanisms linking these two conditions are still poorly understood. 

Several potential mechanisms have been proposed in the etiologies of obese-asthma [9]. Firstly, the excess adipose tissue secretes adipokines (such as leptin and adiponectin) and inflammatory mediators [namely, Interleukin 6 (IL-6) and tumor necrosis factor alpha (TNF-α)] that may affect the airways of susceptible individuals [9]. Diet directly impacts the immune system and may therefore influence both susceptibility to and severity of asthma. Finally, physical activity may be lower in asthmatic and obese individuals. On the other hand, physical activity is a well-studied mechanism in reducing broncholveolar Interleukin 4 (IL-4), Interleukin 5 (IL-5), and eosinophils and in increasing Interleukin 10 (IL-10) [9]. The occurrence of obesity with asthma in children is also reported as a phenotype that usually skews Th1/th2 balance towards Th1, although the type 2-driven asthma is also reported [8]. Nevertheless, these asthma phenotypes represent less precisely defined diseases, and much more information needs to be acquired, especially on the molecular level and the magnitude of influences from nutritional variables in modulating these associations [10]. 

Asthma phenotype in obese individuals is also characterized by more severe symptoms, loss of asthma control, worse quality of life, and different responses to controller medication. However, the underlying mechanisms between these associations are not fully understood [10,11,12]. On the other hand, nutritional inadequacy—high intakes of saturated fat and sodium, higher dietary acid load [13], or low consumption of dietary fiber and micronutrients (namely, vitamin C, E, and carotenoids) involved in antioxidant response—can directly affect airway inflammation and reactivity [14]. Furthermore, as airway smooth muscle proliferative remodeling is one modulator factor for airway hyperresponsiveness, high ingestion of long-chain fatty acids might be a possible risk factor for asthma development by acting as ligands for the free fatty acid receptor 1 (FFAR1) [15]. Therefore, these nutritional aspects may modulate the association between asthma and obesity [16,17]. In addition, dietary acid load, which consists of the difference between endogenously produced non-volatile acid and absorbed alkali precursors [18], has been associated with higher body mass index values (BMI) and metabolic acidosis [19]. On the other hand, potential renal acid load has been widely used in research and is often associated with insulin resistance in humans in type 2 diabetes development [20], hypertension [21], peripheral arterial disease [22], serum uric acid, and development of hyperuricemia [23].

Considering that lungs play an important role in systemic pH and acid–base regulation [24], are a key organ in asthma development, and that nutritional inadequacy in several components and high dietary acid load can affect airway inflammation and reactivity, we aimed to test the hypothesis that dietary acid load may be associated with asthma in children. 

## 2. Participants, Study Design, and Methods

This study included school children from a cross-sectional study assembled in Porto, Portugal. The 20 schools with the highest number of students were selected from 53 primary schools. The evaluations included a questionnaire and a physical and clinical assessment of children. The University Health Ethics Committee approved the study (ARIA 248-13), and informed consent was obtained from the children’s legal guardians. All research was performed in accordance with the Declaration of Helsinki.

### 2.1. Participants

In total, 1602 children (7–12 years old) in the 3rd and/or the 4th grades were invited to participate. Of those, 686 did not return the signed informed consent form, and 58 refused to perform clinical tests. From our initial sample of 858 children, 159 were excluded due to incomplete nutritional information. Thus, this study was based on data from 699 children.

### 2.2. Questionnaire

The evaluation included an ISAAC (The International Study of Asthma and Allergies in Childhood) based questionnaire [25] filled out by the parents, including information on social, demographic, and behavioral characteristics and questions regarding the respiratory/allergic health of the children. Parental education level was accessed and used as socioeconomic status and was recorded as the number of successfully completed years of formal schooling, and children were classified according to the parent with the higher education level. Parental education level was categorized into three classes: ≤9 years; ≥10 years and ≤12 years; and >12 years. Physical activity was defined based on a positive answer to the question, “Does your child participate in any sport activity outside of normal school-period at least once per week?”and then quantifying the physical activity in “less than 2 times a week”, “2–3 times a week”, and “more than 4 times a week”. Food intake was recorded using the interviewer-administered 24 h dietary recall method where participants were questioned accurately about their food and drinks consumption, even reporting brands and consuming time and place [26]. 

### 2.3. Participants Assessments

Assessments were performed at each primary school by a research nurse. Weight (kg) and body fat (%) were measured using a digital scale (Tanita™ BC-418 Segmental Body Analyzer) and height in centimeters (cm) with a portable stadiometer. Body mass index was calculated using the ratio of weight/height2 (Kg/m2) and classified according to the age- and the sex-specific percentiles defined by the World Health Organization (WHO). This study considered 2 categories of BMI: zscore ≤1, indicating non-overweight/obese and ≥zscore >1, indicating overweight/obese. 

Lung function and airway reversibility were assessed by spirometry according to American Thoracic Society (ATS)/ The European Respiratory Society (ERS) (ATS/ERS) guidelines [27] using a portable spirometer (MIR Spirobank, A23-04003237) and were recorded before and after 15 minutes of inhalation of 400 μg of salbutamol. The predictive values for the spirometry parameters were calculated based on the The Global Lung Function Initiative (GLI) 2012 reference data [28] using the GLI-2012 Excel Sheet Calculator [29]. Asthma was defined by at least a 12% and over 200 mL increase in forced expiratory volume in 1 second (FEV1) after bronchodilation or self-reported medical diagnosis with reported symptoms (wheezing, dyspnea, or dry cough) occurring in the previous year.

### 2.4. Potential Renal Acid Load (PRAL) Estimation

The software Food Processor^®^, (ESHA Research, USA) was used to convert food into nutritional data. The macro- and the micronutrients in this study were corrected for total energy intake by normalizing all intakes of macro- and micronutrients to a daily diet of 2136 kcal by regression analysis of the residual method [30]. Potential renal acid load (PRAL) was used to study the dietary acid load and was calculated with the algorithm of Remer et al. [31]: PRAL (mEq/day) =0.49 × protein intake (g/d) + 0.037 × phosphorus (mg/day) − 0.021 × potassium (mg/day) − 0.013 × calcium (mg/day) −0.026 × magnesium (mg/day). 

### 2.5. Data Analysis

The SPSS statistical package software v25.0 (IBM, USA) was used to statistically analyze the data. The Kolmogorov–Smirnov test was used to check continuous variables for normality. The Mann–Whitney test was used to compare variables between sexes. Significant differences were defined with an α-value of less than 5% (*p* < 0.05). Separate binary logistic regression models were fitted for all samples and stratified for body mass categories considering the different immunometabolic links with asthma to estimate the magnitude of the association between PRAL (continuous predictor) and asthma after adjusting for sex, age, energy intake, parent’s education level, and physical activity. The following models were analyzed: a crude model for the main effect (model 0); a model adjusted for energy intake (model 1); a model adjusted for energy intake, sex, and age (model 2); and a model adjusted for energy intake, sex, age, parent’s education level, and physical activity (model 3). Results of the described binary logistic regression analyses are presented as odds ratios (OR) and 95% confidence intervals (95% CI) and are modeled per interquartile range (IQR) increase in PRAL.

A number of further sensitivity analyses were performed to confirm the robustness of our findings. Firstly, the analysis was remade by stratifying the sample with all the body mass index groups defined by the WHO instead of overweight/obese and non-overweight/obese. Secondly, we repeated our analysis using net endogenous acid production (NEAP) equations [32] by stratifying our sample into non-overweight/obese and overweight/obese participants. 

## 3. Results

The median age of the participants was nine, and 52% were girls. The median PRAL was 13 mEq/day, and no differences were found between boys and girls for this variable (Table 1).

When assessing the association between PRAL and asthma in all samples and after categorization according to overweight status, positive and significant associations were found only in children who were overweight/obese (model 0, OR = 1.644, 95% CI = 1.020, 2.650, Table 2), and this association remained significant after adjustment for possible confounders (model 3, OR = 1.953, 95% CI = 1.024, 3.730) (Table 2).

### Sensitivity Analysis

The first sensitivity analysis resulting from stratifying our sample to all the body mass index categories analysis led to stronger associations for overweight children, but the positive associations for the obese individuals were no longer significant (*p* = 0.610). (Supplementary Table S1). 

The analysis using NEAP, whose median value in our sample was 52.57 mEq/d and interquartile range increase was 27.41 mEq/d, also showed a positive association with asthma in overweight/obese individuals, but no significant results were found (*p* = 0.133). (Supplementary Table S2).

## 4. Discussion

We found that, for an increase of 22.04 mEq/d in the potential renal acid load, there was a 95% increase in the chance of asthma, but only among children who were overweight/obese. This association was not significant among non-overweight/obese children. Therefore, our work suggests that dietary acid load might be a nutritional variable to consider in the overweight/obese-asthma phenotype in children. To our knowledge, this is the first work to explore this association with asthma in children both with and without overweight/obesity. 

Mechanisms to explain the findings in overweight/obese children from the present study are not known. The etiology of overweight/obesity and asthma is very complex, and both diseases may share genetic and environmental conditions. However, the interaction between these two conditions is not well defined, although some longitudinal studies suggest that obesity may precede asthma [33,34,35,36]. Dietary acid load tends to induce a chronic low-grade metabolic-acidosis, which is associated with metabolic alterations and obesity prevalence in adults [37,38] as well as with the risk for generation of systemic acidosis and its metabolic consequences in children [39]. Food intake and subsequent metabolism is a complex process that depends on dietary patterns adopted by the individual. Furthermore, diet and some food constituents have a well-described role in acid–base balance due several factors: (1) the chemical composition of foods, mainly protein, phosphorus, sodium, potassium, calcium, and magnesium content; (2) the differences in the absorption rates of these specific nutrients at the intestinal level; (3) the metabolic generation of sulphate from sulphur-containing amino acids; and (4) the ionic valence of calcium and magnesium [40]. 

In the present study, median PRAL value (median = 13.1; 25th; 75th percentile = 1.51; 15.57) was higher than others found in cohort studies (median = 6.63; 25th; 75th percentile = −0.36; 14.56) in children [41]. This value is typically associated with an excess intake of acid precursors, and PRAL with negative values indicates an ingestion of base-forming potential foods that are nearly exclusively found in vegetables and fruits groups [42]. However, no differences for PRAL were found between BMI categories in each sex. Generally, most proteins produce acid, and fruits/vegetables produce alkali precursors. Recent data showed that a typical western dietary pattern produces 50–100 mEq H^+^ per day that need to be excreted from the body in order to keep homeostasis [43] because, under normal conditions, blood pH is maintained in a narrow physiological range, and dietary acid load needs to be neutralized [20]. Considering the large amount of H^+^ produced within a typical western diet, situations of chronic mild metabolic acidosis have been considered. Additionally, high dietary acid loads usually result in a high net production of volatile acids such as hydrogen chloride (HCl) and hydrogen sulphate (H_2_SO_4_). The physiological way of excreting these compounds is by expelling carbon dioxide (CO_2_) via the lung and by the excretion of produced sodium salts from the non-volatile acids by the kidneys in the forms of ammonium and titratable acid [20,44]. As the lung plays main roles in both asthma development and in the dietary acid load buffering system, we hypothesized that high dietary acid load can be a plausible mechanism in asthma occurrence or development risk in obese children that, due to being overweight/obese, tend to have a different immunometabolic stress compared to non-overweight/obese peers. 

The metabolic acidosis caused by a typical western diet with a high dietary acid load also results in an imbalance in the supply of nutrient precursors of HCO_3_ and H^+^. Daily HCO_3_ delivery to the systemic circulation may lag behind that of H^+^. Specifically, the rate of endogenous generation of HCO_3_ from the metabolism of dietary inorganic, predominantly potassium salts of organic acids (e.g., potassium citrate, malate) fails to keep pace with the rate of endogenous generation of H+ from non-carbonic acids (e.g., sulfuric acid, various organic acids) that can appear either as end-products of metabolism or ingested precursors. Thus, dietary bicarbonate deficiency may be driven by a typical high dietary acid load diet, because there is insufficient intake of dietary plant food rich in potassium-coupled bicarbonate precursors [45]. This may be another important factor associated with our proposal mechanism, because lower levels of serum bicarbonate and higher levels of anion gap were associated with lower cardiorespiratory fitness in adults [46].

Our study has a few limitations. The cross-sectional design does not allow the establishment of causal relationships between dietary acid load and asthma development. Additionally, physical activity was defined based on a single question using self-reported data, which may not reflect the real activity performed by children and may lead to bias. However, compared to direct measures, self-reported methods may be able to represent the type of activity that is undertaken [47]. Although current asthma was not diagnosed by a physician, the used criteria in this study included a standardized operational definition of asthma supported by objective lung function measurements [48]. Another limitation of our study is the fact that food intake was evaluated using questionnaires, which can lead to bias. Lastly, although several confounders were considered, we cannot rule out that other unknown or unmeasured confounders may have important influences on our findings. Additionally, the low number of obese children did not allow us to extrapolate our results to the obese-asthma phenotype. Therefore, further studies should address this topic.

Our study also has important strengths. To our knowledge, this study is the first community-based study performing the evaluation of the association between dietary acid load and asthma. Thus, it is also the first work to suggest a plausible mechanism and link between a dietary factor that can be modulated by food intake and asthma development in overweight/obese individuals. Additionally, BMI was calculated based on measured height and weight, avoiding self-perceptions of weight categories by parents, since most parents underestimated their children’s overweight/obese status [49]. Furthermore, a comprehensive clinical assessment on a large number of participants, including anthropometry and lung function assessment, was performed, and PRAL was used to assess dietary acid load; PRAL is a score commonly used in epidemiologic studies based on protein, phosphorous, potassium, calcium, and magnesium intake and is a validated tool in children [41,50].

## 5. Conclusions

In conclusion, we showed, for the first time, that asthma and dietary acid load are associated in overweight/obese children. These findings highlight the importance and the health benefits of a diet rich in base-yielding vegetables and fruits to balance dietary acid load and maintain homeostasis. Moreover, the obese-asthma phenotype is traditionally a more difficult to control asthma phenotype, with mechanisms remaining poorly known. This newly suggested link in the obese-asthma phenotype may lead to a dietary solution that could prevent asthma in overweight/obese individuals.

## Figures and Tables

**Table 1:** Characteristic of the participants.

Characteristics	Total *n* = 699	Girls *n* = 365	Boys *n* = 334	*p*	Girls (*n* = 365)		*p*	Boys (*n* = 334)		*p*
					Non-overweight/obese	Overweight/obese		Non-overweight/obese	Overweight/obese	
**Age (years)**	9.0 (8.0;9.0)	9.0 (8.0;9.0)	9.0 (8.0;9.0)	0.465	9.0 (8.0;9.0)	9.0 (8.0;9.0)	0.163	9.0 (8.0;9.0)	9.0 (8.0;9.0)	0.719
**Parental education [n (%)]**				<0.001			0.650			0.467
**0–9 years**	174 (24.9)	131 (30.2)	43 (16.2)		53 (32.5)	32 (29.9)		56 (31.1)	31 (37.8)	
**10–12 years**	164 (23.5)	112 (25.8)	52 (19.6)		48 (29.4)	32 (29.9)		60 (33.3)	23 (28.0)	
**>12 years**	197 (197)	92 (21.2)	105 (39.6)		62 (38)	43 (40.2)		64 (35.6)	28 (34.1)	
**Asthma [n (%)]**	68 (9.7)	48 (11.1)	20 (7.5)	0.129	21 (9.7)	16 (12.7)	0.385	13 (5.7)	7 (6.7)	0.715
**Physical activity [n (%)]**				0.075			0.492			0.956
**Less than 2 times a week**	301 (43.1)	193 (44.5)	108 (40.8)		100 (52.9)	64 (56.1)		91 (44.0)	38 (40.4)	
**2–3 times a week**	247 (35.3)	156 (35.9)	91 (34.4)		67 (35.4)	40 (35.1)		76 (36.7)	42 (44.7)	
**More than 4 times a week**	78 (11.2)	38 (8.8)	40 (14.1)		22 (11.6)	10 (8.8)		40 (19.3)	14 (14.9)	
**Energy intake (kcal/day)**	2135.8 (1782.2;2471.1)	2049.8 (1721.1;2499.6)	2214.0 (1941.9;2591.9)	<0.001	2059.1 (1742.7;2379.7)	2065.4 (1721.4;2402.8)	0.830	2194.3 (1914.8;2545.0)	2263.2 (2013.2;2624.9)	0.246
**Protein (g/day)**	89.2 (71.1;114.6)	109.0 (87.0;110.8)	95.4 (76.3;95.4)	0.005	86.0 (71.8;109.1)	95.0 (73.0;112.4)	0.242	91.7 (75.1;113.5)	103.6 (85.4;121.1)	0.013
**Phosphorus (mg/day)**	1352.1 (899.8;1352.3)	1304.0 (1070.4;1582.3)	1389.1 (1149.9;1685.5	0.014	1325.4 (1099.5;1569.1)	1352 (1118.7;1673.3)	0.453	1401.8 (1160.4;1665.1)	1498.0 (1160.7;1658.5)	0.222
**Potassium (mg/day)**	3012.7 (2415.5;3703.8)	2989.4 (2389.3;3677.4)	3014.2 (2478.7;3764.8)	0.263	3003.3 (2377.8;3675.5)	3059 (22356.3;3753.9)	0.914	3120.9 (2529.9;3775.3)	3198.7 (2524.5;3911.3)	0.820
**Magnesium (mg/day)**	261.7 (216.3;318.3)	256.2 (208.5;314.5)	272.9 (223.8;320.3)	0.067	256.8 (216.7;310.6)	266.5 (209.9;319.2)	0.730	272.0 (228.0;321.4)	281.4 (221.6;334.4)	0.985
**Calcium (mg/day)**	912.3 (647.0;1164.9)	900.5 (641.9;314.5)	921.9 (666.9;1148.9)	0.791	917.3 (658.4;1173.4)	896 (638.9;1184.7)	0.630	900.5 (644.8;1161.2)	942.5 (698.8;1226.6)	0.510
**PRAL (mEq/day)**	13.1 (1.51;15.57)	14.0 (1.8;26.2)	11.5 (0.53;23.4)	0.138	13.4 (3.2;23.4)	13.4 (1.7;27.7)	0.501	13.8 (0.88;27.9)	10.4 (-4.66;10.4)	0.133
**BMI classification [n (%)]**				0.886						
**Underweight**	8 (1.1)	4 (1)	4 (1.5)							
**Normal weight**	412 (58.9)	247 (62.5)	163 (62.2)							
**Overweight**	156 (22.3)	94 (23.8)	53 (20.2)							
**Obese**	92 (13.2)	50 (12.7)	42 (16.0)							

^1^ Data reported as median (25th; 75th percentile) unless otherwise stated. PRAL: potential renal acid load; BMI: body mass index; WHO: World Health Organization.

**Table 2:** Logistic regression analysis for the association between PRAL and asthma for all samples and according to overweight/obese status ^a^.

		OR	CI (95%)	*p*
All participants				
PRAL				
	Model 0	1.171	0.900, 1.525	0.241
	Model 1	1.123	0.832, 1.516	0.447
	Model 2	1.116	0.826, 1.510	0.475
	Model 3	1.213	0.863, 1.703	0.266
Non-overweight/obese				
PRAL				
	Model 0	0.916	0.651, 1.288	0.613
	Model 1	0.912	0.628, 1.324	0.628
	Model 2	0.910	0.627, 1.323	0.622
	Model 3	0.959	0.617, 1.491	0.853
				
Overweight/obese				
PRAL				
	Model 0	**1.644**	1.020, 2.650	0.041
	Model 1	**1.874**	1.022, 3.337	0.042
	Model 2	**1.820**	1.003, 3.300	0.049
	Model 3	**1.953**	1.024, 3.730	0.042

^a^ OR and 95% CI modeled per interquartile range increase in PRAL (22.04 mEq/d) OR: odds ratio. Model 0—unadjusted model, Model 1—adjusted for total energetic value, Model 2—adjusted for energetic value, gender, and age, Model 3—adjusted for energy intake, sex, age, parent’s education level, and physical activity. Significant results are shown in bold.

## Supplementary Materials

The following are available online at https://www.mdpi.com/2072-6643/11/9/2255/s1, Table S1: logistic regression analysis for the association between PRAL2 and asthma for all sample and according overweight/obese status ^a^, Table S2: logistic regression analysis for the association between NEAP2 and asthma for all sample and according overweight/obese status ^a^.
