# Supplementary material for: Dietary Acid Load: A Novel Nutritional Target in Overweight/Obese Children with Asthma?
Source: Nutrients. 2019 Sep 19;11(9):2255. doi: 10.3390/nu11092255 (PMC6770083; doi:10.3390/nu11092255)
Supplement: Supplementary file 1 [file nutrients-11-02255-s001.pdf]

Supplementary Table S1. logistic regression analysis for the association between PRAL[2] and asthma for all sample and according overweight/obese status [a].

			OR	CI (95%)	p
Normal weight					
	PRAL				
		Model 0	0.916	0.651,1.288	0. 613
		Model 1	0.912	0.628,1.324	0.628
		Model 2	0.910	0.627,1.323	0.622
		Model 3	0.959	0.617,1.491	0.853
Overweight					
	PRAL				
		Model 0	**1.841**	1.056,3.208	0.031
		Model 1	**2,573**	1.195,5.541	0.016
		Model 2	**2,536**	1.184,5.433	0.017
		Model 3	**2.682**	1.164,6.178	0.021
Obese					
	PRAL				
		Model 0	1.268	0.433,3.718	0.665
		Model 1	1.061	0.304,3.702	0.926
		Model 2	1.184	0.330,4.244	0.795
		Model 3	1.569	0.278,8.841	0,610

[a] OR and 95% CI modeled per interquartile range increase in PRAL (22.04 mEq/d) [2]PRAL: Potential renal acid load; OR = Odds ratio. Model 0 – Unadjusted model, Model 1- adjusted for total energetic value, Model 2- adjusted for energetic value, gender and age, Model 3 - adjusted for energy intake, sex, age, parent's education level and physical activity. Significant results in bold.

Supplementary Table S2. logistic regression analysis for the association between NEAP[2] and asthma for all sample and according overweight/obese status [a].

		OR	CI (95%)	p
All participants				
NEAP				
	Model 0	1.055	0.800,1.391	0.706
	Model 1	1.000	0.710,1.409	0.999
	Model 2	0.941	0.699,1.393	0.941
	Model 3	1.017	0.703,1.470	0.930
Non Overweight/obese				
NEAP				
	Model 0	0.906	0.617,1.330	0.614
	Model 1	0.808	0.506,1.289	0.371
	Model 2	0.803	0.502,1.283	0.359
	Model 3	0.793	0.456,1.379	0.412
Overweight/obese				
NEAP				
	Model 0	1.178	0.754,1.842	0.472
	Model 1	1.545	0.854,2.793	0.150
	Model 2	1.525	0.836,2.783	0.169
	Model 3	1.632	0.861,3.093	0.133

[a] OR and 95% CI modeled per interquartile range increase in NEAP (27.41 mEq/d) [2]NEAP: Net endogenous acid production ; OR = Odds ratio. Model 0 – Unadjusted model, Model 1- adjusted for total energetic value, Model 2- adjusted for energetic value, gender and age, Model 3 - adjusted for energy intake, sex, age, parent's education level and physical activity. Significant results in bold.
